# Historical roots of histrionic personality disorder

**DOI:** 10.3389/fpsyg.2015.01463

**Published:** 2015-09-25

**Authors:** Filipa Novais, Andreia Araújo, Paula Godinho

**Affiliations:** ^1^Neurosciences and Mental Health Department, Santa Maria Hospital, Lisbon, Portugal; ^2^Child and Adolescent Psychiatry Department, Hospital Dona Estefânia, Lisbon, Portugal

**Keywords:** hysteria, histrionic, personality disorder, history, historical roots, neurology

## Abstract

Histrionic Personality Disorder is one of the most ambiguous diagnostic categories in psychiatry. Hysteria is a classical term that includes a wide variety of psychopathological states. Ancient Egyptians and Greeks blamed a displaced womb, for many women’s afflictions. Several researchers from the 18th and 19th centuries studied this theme, namely, Charcot who defined hysteria as a “neurosis” with an organic basis and Sigmund Freud who redefined “neurosis” as a re-experience of past psychological trauma. Histrionic personality disorder (HPD) made its first official appearance in the Diagnostic and Statistical Manual of Mental Disorders II (DSM-II) and since the DSM-III, HPD is the only disorder that kept the term derived from the old concept of hysteria. The subject of hysteria has reflected positions about health, religion and relationships between the sexes in the last 4000 years, and the discussion is likely to continue.

## Introduction

Histrionic personality disorder (HPD) is the only modern category in diagnostic classifications that conserved a derivative of the old concept *Hysteria* (Sulz, 2010). Several psychiatric disorders derived from the original term hysteria such as the conversion disorder, the somatization disorder, somatoform disorders, phobic anxiety, the term mass hysteria, and finally the HPD. Although different authors extensively studied this theme across time, the authors will focus on HPD.

The word *hysteria* derived from the Greek term “hystera,” meaning the womb or uterus. It has been used since ancient times and appears in texts of the Egyptians, Greeks, and Romans. Since then, the meanings of *hysteria* have mirrored the preoccupations of the societies at each time.

In the old Rome, the word “Histrione” was already used to define the actors that represented coarse farces representing those who are false and theatrical (Zimmerman, 1999).

## From Egyptians to Hippocrates

The oldest record is an Egyptian medical papyrus dating from around 1990 BC, the *Kahun Papyrus*, which is the first known medical gynecological text. Plato (429 ± 347 BC) described this phenomenon as “The animal within them is desirous of procreating children, and when remaining unfruitful…gets discontented and angry, and wandering in every direction through the body…drives them to extremity, causing all varieties of disease…” (Illis, 2002). Many women’s afflictions, including choking, mutism, and paralysis were attributed to a condition called the “wandering womb” or “the wicked womb.” It was Hippocrates (460 ± 377 BC) who first introduced the term “Hysteria” and described it as the consequence of a dry womb rising toward the throat searching for humidity, thereby impeding breathing. The neurotoxic effects of the “frustrated uterus” would affect widows and virgins (Bogousslavsky, 2011). Galen (AD 129 ± 216 BC) instead blamed the blocked menstrual flow and sexual abstinence. One of his most striking views was that men could also suffer hysterical symptoms caused by retained sperm. His ideas contributed to initiate a debate, which had run for centuries, over whether men could or not have hysteria. Galen’s views persisted among the medical practitioners of Christian post-Roman Britain (Edwards, 2009).

## From Middle Ages to the 18th Century

During Middle Ages, as the attitude toward sickness changed from naturalistic to demonotheologic, with Augustine of Hippo (354–430 AC) and other theologians, hysteria came to be seen as a manifestation of demonic possession. Convulsions and the so-called “suffocations of the matrix” were considered as an expression of sexual pleasure and, therefore, sin. Devil could enter women’s body to possess them and the hysterical become the witch, persecuted by the Catholic Church and many of those were killed by the inquisition (Roudinesco and Plon, 2000).

With the renewed interest on empiricism and science during Renaissance, old Greek concepts of hysteria were recuperated. Similar therapies to those prescribed in ancient Greek civilizations, such as genital stimulation by horse riding, dancing and, in particular, marriage and sexual intercourse were still prescribed for such condition (Edwards, 2009).

Several researchers including Charles Lepois, Thomas Willis, Thomas Sydenham, and Pierre Pomme had great interest in the study of hysteria (Teive et al., 2014).

Some of these authors defied the original theories that connected hysteria to the uterus and some defended that the disease was originated in the brain. One of the first was Thomas Willis (1621 ± 1675) who argued that hysterical disorders, the so-called “convulsive distempers,” were caused by an excess of animal spirits carried by the nerves to different parts of the body, introducing a new etiology for the disease. He believed in a nervous origin instead of vapors opening the door to the desexualisation of the disease (Risse, 1988).

The famous clinician Thomas Sydenham, (1624–1689) was one of the most important contributors to the study of hysteria at his time. He published a treatise on hysteria called *Epistolary Dissertation on the Hysterical Affections* and stated that hysteria was the most common of all diseases afflicting both men and women and the more richer and civilized a patient was, the more likely he or she was to be afflicted. One of his most remarkable conclusions was that hysteria could take multiple forms in order to imitate several other diseases, frequently triggered by intense emotions such as anger, grief, terror or passions (Gilman et al., 1993).

William Cullen, (1769), a noted Scottish physician, published *Synopsis Nosologiae Methodicae*, a classification of diseases where hysteria figured on the group called *neuroses*. These diseases were considered to result from nervous system malfunction involving changes in sensibility and motion. Hysteria was included in the class of illnesses characterized by irregular muscular contractions, the so-called spasmodic diseases, but Cullen still admitted that in its origin were gynecological problems (Risse, 1988).

Philippe Pinel, (1745–1826) considered that diagnostic difficulties were associated with the numerous disorders and symptoms attached to it, so he defended the study of hysteria in its uncluttered or “pure state.” He included hysteria in his “*Nosographie Philosophique*” (1813) placing it in the group called “Neuroses” (Whitaker et al., 2007).

During this period, hysteria was a serious subject in medical schools and textbooks. Some authors considered it to reflect psychological frustrations directly linked to the restricted role of women in society (Risse, 1988). Griesinger, (1817–1868) kept the view that hysteria was related to genital disorders and sexual frustration but also involving “morbid action of… the brain” (Gilman et al., 1993).

In 1859 Pierre Briquet, (1796–1881) published his “*Traité Clinique et Therapeutique de L’Hysterie*” presenting data from 430 hysterical patients collected in 10 years. He rejected the idea of the uterine origin of the disease and considered it as a “neurosis of the brain” in someone of the “hysterical type.” Briquet had a remarkable contribution for development of the HPD; he considered this type of personality traits as the ground for the development of the histrionic disorders (Mai and Merskey, 1981). He introduced sociological and material concepts in the comprehension of hysteria, such as living and working conditions. The industrialization, with the development of the trains and the subsequent numerous traumatic accidents, brought up the discussion about the hysteria in men. Between 1880 e 1900, hysteria was epidemic: writers, doctors and historians agreed to refer to the industrial social crises, like strike, as a sign of the feminine convulsive nature and frequently applied the terms hysteria and “uterine furies” to designate them (Roudinesco and Plon, 2000).

At the end of the 19-century, Salpêtrière Hospital acquired a remarkable importance on the study of hysteria and hypnotism due to the famous French neurologist, Jean-Martin Charcot (1825 ± 1893), who created the study of Diseases of the Nervous System there, in 1862. He had many remarkable collaborators such as Albert Pitres, Paul Richer, Georges Gilles de la Tourette, Paul Sollier, Joseph Babinski, Sigmund Freud, and Pierre Janet creating the famous Salpêtrière’ s School of Neurology. His interest on hysteria probably started after 1870, when Charcot’s took charge of the Delasiauve service, a place where mainly epileptics and hysterics were admitted (Bogousslavsky et al., 2009). Using a photographic camera, after long and detailed observations and methodical comparisons of hysteria with other conditions, he considered two main forms of hysteria—with and without convulsions. The hysteroepilepsy or “*grandes crises d’hystérie*” were described as having four stages: 1. Epileptoid; 2. Contortions and acrobatic postures (Clownism); 3. Emotional gestures (“*attitudes passionnelles*”); and 4. Final delirium (Teive et al., 2014). Charcot considered hysteria as a “neurosis” with an organic basis and described permanent clinical features in patients who were also prone to paroxysmal fits, the “stigmata”: sensory dysfunction, hyperexcitability and visual field narrowing (Bogousslavsky et al., 2009).

According to him, the presumed neurological impairment was dynamic in nature and produced by unconscious mental processes (Macmillan, 1997). Hysterical symptoms occurred in genetically predisposed individuals and were manifested within familiar circumstances. Therefore, he stated that a fundamental condition of the treatment should be the isolation from family members and called this “the moral or mental side of treatment” (Illis, 2002).

One of Charcot’s most remarkable students was Babinski, (1857–1932) who defined hysteria as a psychic state that would give the patient the ability of “auto-suggestion,” so that the patient would be able “to be persuaded” and therefore was prone to “healing” by suggestion (Philippon and Poirier, 2009). Consequently he recommended the term “pithiatism” (from the Greek: created by suggestion and curable by persuasion). Despite the influences of his master, he presented his own theory about hysteria, as well as several approaches and specific criteria in order to differentiate organic from hysterical symptomatology (Mai, 2004; Allilaire, 2007; Clarac et al., 2008).

Later, Charcot introduced hypnosis as a therapeutic technique and also as an experimental tool to the study of hysterical phenomena and its underlying neurophysiology and psychogenic trauma-related mechanisms of the hysterical neuroses (Levin, 1978).

The principles of hypnosis have been previously established by Franz Anton Mesmer, (1734–1815) who created the so-called “Animal magnetism,” a pervasive property of nature that could be used as an effective therapy for a wide variety of conditions and its therapeutically application—the “mesmerization” (Lanska and Lanska, 2007).

Charcot and his group have been criticized by the School of Nancy and his main investigator Hippolyte Bernheim, (1840–1919), a French physician and neurologist. While Charcot believed that hypnosis was based on physiologically well-determined phenomenon only applied, as a therapeutic and diagnostic technique, to hysterical patients, Bernheim proposed that it was based on changes in psychological functioning; different features of hypnosis would therefore reflect different degrees of suggestibility. He also argued that suggestibility was a normal human trait and not an abnormal phenomenon as Charcot defended (Macmillan, 1997).

Both Bernheim and Charcot had important influences on Sigmund Freud’s, (1856–1939) latter theories. Freud, who later developed the psychoanalytic theory leading to the redefinition of hysteria and the creation of different syndromes that came from the original concept, went to the famous Salpêtrière in October 1885 in order to study with Charcot. He started translating some of Charcot’s lectures and defending his views. 2 years after, he translated Bernheim’s work and visited Nancy in the summer of 1889 (Macmillan, 1997).

Another important author that had influenced the work of Freud was Pierre Janet, (1859–1947), also a Charcot follower. Many of Freud’s basic concepts were developed or elaborated by Janet, such as psychological automatism, consciousness, subconsciousness, narrowed field of consciousness, dissociation, suggestibility, fixed idea, and emotion (Hart and Horst, 1989). Janet considered that hysteria results from the idea the patient has about pathology, translating it into a physical disability. He studied five hysteria’s symptoms: anesthesia, amnesia, abulia, motor control diseases, and modification of character (Tasca et al., 2012).

In 1895, Freud and Breuer, (1842–1925) published the “Studies on Hysteria,” including the famous case study of Anna O and the formulation of three types of hysteria: defense, retention and hypnoid hysteria (Breuer and Freud, 1955). Freud defied the traditional idea that defended that hysteria was caused by the lack of conception and motherhood, proposing that hysteria was a disorder caused by a lack of libidinal evolution (setting the stage for the Oedipal conflict), so the consequence, and not the cause, would be the lack of conception as a result of the incapacity of the hysterical to live a mature relationship. Hysterical symptoms would therefore be the expression of the impossibility of fulfillment of the patient’s sexual drive. Freud also added, to this paradigm, the concepts of “primary benefit” and “secondary advantage” associated with the use of these symptoms to satisfy patient’s needs (Tasca et al., 2012). This new paradigm concerning the emotional origin of hysterical symptoms was often applied to shell shock and other “war neurosis” during the World War I and II (Crocq and Crocq, 2000). In fact, with the war and after that, during the 1940s and the 1950s, the interests in this matter grew rapidly.

Freud explored traumatic experiences occurring in the family in order to provide an explanation for hysteria. Unacceptable feelings connected to seduction were repressed and converted into somatic symptoms. Latter he found that many of these reports were false, so he concentrate on intrapsychic factors. Patients repressed not actual happenings but their own sexual fantasies (Slipp, 2014).

## Histrionic Personality Disorder

Although the roots of modern histrionic personality can be traced back to Freud’s description of “hysterical neuroses” (Sperry, 2003), personality was already a matter of attention before.

In the mid-19th century, Ernst von Feuchtersleben, (1765–1834) who wrote the Textbook of Medical Psychology (1845) made the first psychosocial description of what would become the histrionic personality. He described hysterical women as being sexually heightened, selfish and “overprivileged with satiety and boredom” (Millon, 2011).

Ernst Kretschmer, (1888–1964), a German psychiatrist known for the establishment of a typology based on the human constitution, suggested that hysterics show “a preference for what is loud and lively, a theatrical pathos, an inclination for brilliant roles…(and) a naïve, sulky egotism” (Bornstein et al., 2015). Another Kretschmer’s important contribution was the demand for objective criteria in order to distinguish hysteria from simulation (Lerner, 2003).

The first providing a detailed psychoanalytic description of the hysterical personality style was Wilhelm Reich, (1897–1957), an Austrian psychoanalyst. He wrote “coquetry in gait, look or speech betrays, especially in women, the hysterical character type…We find fickleness of reactions…and…a strong suggestibility, which never appears alone but is coupled with a strong tendency to reactions of disappointment…”

A decade after his work, Otto Fenichel, (1897–1946), a psychoanalyst of the so-called “second generation,” added another characteristic to this description: the pseudo-hypersexuality, noting that these individuals “are inclined to sexualize all nonsexual relations…” (Bornstein et al., 2015).

Easser and Lesser, (1965) seek to integrate two different earlier approaches: the ego psychology school and Freud’s libido theory. They proposed a classification of hysterics consisting on two extremes—the hysterical personality and the “hysteroid” (borderline) personality. Zetzel, (1968) also divided patients into “good” hysterics, who function well, and “bad” hysterics, who have weak egos and poor object relations. This latter group of patients has a profile and level of functioning similar to the one seen in borderline patients (Slipp, 2014).

Several theorists studied the particular traits of this type of personality including histrionic’s impressionist cognitive style and inattention to detail. In his book, “Hysterical Personality Style and the Histrionic Personality Disorder,” Horowitz (1991), focused on the connection between perception and behavior in histrionic personality; he argued that it was based on an underlying information processing bias. A disturbed mental representation of the self would constitute the link to the various features of this type of character. On the other hand, according to the biosocial-learning model, proposed by Theodore Millon and other authors, this personality type may arise from unconscious patterns of reinforcement provided by parents and others (Blaney et al., 2015). The cognitivists Beck et al. (2004) suggested that histrionic person believe that potential caregivers are not trustful and should be manipulated instead. According to these authors, their core believes include “I am inadequate and unable to handle the life on my own” and “It is necessary to be loved by everyone, all the time.”

Since the first attempts to the establishment of diagnostic criteria in hysteria, there has been considerable controversy, considering the etiology, the definition and even the existence of such condition.

## Discussion and Conclusion

The terms hysteria, hysterical personality, and HPD mark the development of unceasing attempts to identify a distinct pattern of psychopathology (Bakkevig and Karterud, 2010).

The first edition of the American Diagnostic and Statistical Manual (DSM-I), published in American Psychiatric Association (1952), had no category for hysterical personality although some of its features were included in the “emotionally unstable personality.” The DSM-II (American Psychiatric Association, 1968) was strongly impacted by psychoanalysis: some personality disorders had to be differentiated from other neuroses with the same name (e.g., hysterical, obsessive-compulsive, and neurasthenic personalities and neuroses). Following the medical model created by Emil Kraepelin, in DSM-III (American Psychiatric Association, 1980), and the subsequent DSM-III-R (American Psychiatric Association, 1987) and DSM-IV (American Psychiatric Association, 1994), personality disorders were described as discrete types and grouped into three clusters. The term *hysterical* from DSM-II was replaced with “histrionic” in DSM-III following the proposition of Paul Chodoff who considered pejorative the description of the “hysterical female” as labile, egocentric, seductive, frigid and childish, as described in his article “The diagnoses of hysteria: An overview” (Chodoff, 1974). From DSM-III to DSM-IV-TR, (American Psychiatric Association, 2000) diagnostic criteria of HPD had several changes mainly due to the argument of “unspecificity.” An important change occurred from DSM-III-R to DSM-IV:5 criteria were considered the threshold for obtaining the diagnosis, as compared to 4 criteria in DSM-III–R. This lead to a decline in the number of patients diagnosed with HPD (Blais and Baity, 2006).

The merits of compounding typological and dimensional models of personality were questioned during the preparation of DSM-5, reopening a century-old debate (Crocq, 2013). Data, although sparse, actually suggest that the rate of presentation of “hysteria” in neurological practice has remained stable over time (Stone et al., 2008).

Bakkevig and Karterud (2010), in a study carried out with a sample of patients attending psychiatric day hospital, concluded that the prevalence of HPD was very low (0.4%) and comorbidity was high, especially with borderline, narcissistic, and dependent personality disorders. They suggested that the HPD category should be deleted from the DSM system, excepting that clinical phenomena of exhibitionism and attention-seeking, which are the dominant personality features of HPD, should be preserved in an exhibitionistic subtype of narcissism. Nevertheless, HPD remains present in DSM-5 (American Psychiatric Association, 2013).

Edwards (2009) advocates that those who argue that hysteria has disappeared from clinical practice, “miss the point” and “that it has merely changed to reflect the preoccupations of our society…”

Concerns with stigma and lack of specificity of the term hysteria, and its derivative histrionic, led to its residual presence in modern classifications but the theme and its modern diagnosis that emerged from the original concept kept their topicality and importance in clinical practice.

For about 4000 years the construct of hysteria and its derivatives has reflected attitudes about health, religion and relationships between the sexes and the interest raised by this condition is likely to continue (Illis, 2002).

### Conflict of Interest Statement

The authors declare that the research was conducted in the absence of any commercial or financial relationships that could be construed as a potential conflict of interest.
